# A host subtraction database for virus discovery in human cell line sequencing data

**DOI:** 10.12688/f1000research.13580.3

**Published:** 2019-05-21

**Authors:** Jason R. Miller, Kari A. Dilley, Derek M. Harkins, Timothy B. Stockwell, Reed S. Shabman, Granger G. Sutton

**Affiliations:** 1 J. Craig Venter Institute, Rockville, MD, 20850, USA; 2 Shepherd University, Shepherdstown, WV, 25443, USA; 3 National Biodefense Analysis and Countermeasures Center (NBACC), Fort Detrick, MD, 21702, USA; 4 American Type Culture Collection, Gaithersburg, MD, 20877, USA

**Keywords:** RNAseq, human cell lines, HepG2, HuH-7, Jurkat, host subtraction

## Abstract

The human cell lines HepG2, HuH-7, and Jurkat are commonly used for amplification of the RNA viruses present in environmental samples. To assist with assays by RNAseq, we sequenced these cell lines and developed a subtraction database that contains sequences expected in sequence data from uninfected cells. RNAseq data from cell lines infected with Sendai virus were analyzed to test host subtraction. The process of mapping RNAseq reads to our subtraction database vastly reduced the number non-viral reads in the dataset to allow for efficient secondary analyses.

## Introduction

Host subtraction is the bioinformatics process of filtering reads derived from host DNA and RNA ( Daly *et al*., 2015). Host subtraction enriches the non-host component of sequence datasets and is especially attractive for assays involving high-throughput sequencing technologies that generate short reads in high volume, where data reduction can realize cost savings. Following host subtraction, remaining reads can be mapped to references and counted, or used as queries to sequence databases, or possibly assembled to reconstruct novel transcript or genome sequences.

Host subtraction resources are needed for human cell lines that are widely used for RNA virus propagation. This includes three human cell lines Jurkat, HuH-7, and HepG2 commonly used to grow viruses or to amplify viruses suspected to be present in clinical isolates. The Jurkat line, derived from human T cells, supports replication of HIV and some Herpesviruses. Jurkat cells have been described to harbor Xenotropic murine leukemia virus-related virus (XMRV), a now famous gammaretrovirus incorrectly proposed as a causative agent of human prostate cancer and chronic fatigue syndrome ( Cingöz *et al*., 2012). The HuH-7 and HepG2 lines, both derived from liver cells, are widely used for virus research among multiple virus families. These cell lines are permissive to the growth of several RNA viruses. Huh7 cells support Hepatitis C virus (HCV) replication ( Lindenbach *et al*., 2005), though not all Huh-7 lines are permissive to HCV ( Sainz *et al*., 2009). Huh-7 cells are also permissive for Dengue virus ( Lin *et al*., 2000). In addition, Vesicular Stomatitis Virus (VSV), of the *Rhabdoviridae* family, replicates efficiently in HepG2 and HuH-7 ( Marozin *et al*., 2008). HepG2 cells are permissive to Influenza virus ( Ollier *et al*., 2004) and have been shown to harbor cell-line specific chromosomal rearrangements ( Wong *et al*., 2000). Notably, Jurkat, HuH-7, and HepG2 cell lines are all permissive to the Paramyxovirus Sendai virus (SeV). While SeV does not cause disease in humans, infection in mice results in pneumonia in mice ( Faísca & Desmecht, 2007). More importantly, SeV is extensively used as a model to study virus-host interactions since it has a similar genomic organization to pathogenic viruses that include Ebola, Marburg, Hendra and Nipah Virus.

An ideal subtraction resource for any cell line would include a complete genome sequence. Less expensive alternatives are available for cell lines derived from humans. One subtraction alternative is a reference human genome supplemented with a collection of cell-line-specific sequences. To this end, we have developed a sequence subtraction database (SDB) that permits enrichment of viral sequences through the computational depletion of host sequences. We developed an SDB, named SDB1, representing three human tissue cell lines that are often used for the detection of RNA viruses that infect humans. Reducing the size of the NGS dataset by removing host background reads allows the researcher to perform subsequent analyses (e.g. *de novo* assemblies, read mapping, homology searches) more efficiently and with less computational requirements.

## Methods

### Cell line sequencing

Frozen HepG2 cells were obtained from ATCC, part number ATCC HB-8065, lot # 61983117. The HepG2 cell line is derived from a liver hepatocellular carcinoma of a 15-year-old Caucasian male. Frozen HuH-7 cells were obtained from JCRB Cell Bank, part number JCRB0403, lot # 08062010. HuH-7 is described as a well-differentiated human hepato-cellular carcinoma cell line derived from the liver or gallbladder of a 57-year-old male Japanese patient, who died in 1985. Frozen Jurkat cells were obtained from ATCC, part number ATCC TIB-152, lot # 6213515. This Jurkat cell line was derived from peripheral blood of a 14-year-old boy who was diagnosed with acute T cell leukemia. Cells were maintained following the standard recommended protocol per cell line.

A single genomic DNA library was prepared per cell line. Genomic DNA was isolated from the cell line using a Qiagen genomic DNA isolation kit. Bioanalyzer analysis confirmed high molecular weight DNA was recovered. After Blue Pippin size selection, fragments appeared to be 290bp. NextGen paired end barcoded genomic library construction was performed with a NEBNext whole genome library prep kit. NextGen Library quantification and normalization was performed by qPCR. Each library was test sequenced with one run on an Illumina MiSeq, and then sequenced with two runs of an Illumina NextSeq 500 using the Illumina High Output Kit. Reads were demultiplexed, which removed barcodes and sequencing adapters.

Four RNA libraries were prepared per cell line. Cells were either mock infected or infected with SeV as previously described ( Dilley *et al.*, 2017). In each condition, libraries were either treated to deplete ribosomal RNA or total RNA was subjected to library construction. Libraries were multiplexed and sequenced using one run on an Illumina NextSeq 500 using the Illumina Mid Output Kit.

### SDB construction

DNA and RNA sequence reads were trimmed of adapter using CutAdapt 1.8.1. RNA sequence reads were also trimmed of low quality bases, adapter sequences, and resulting short reads using Trimmomatic 0.35. The DNA read sets were not filtered based on length, quality, or number of ambiguous base calls.

Reads were mapped with bowtie2 ( Langmead & Salzberg, 2012) version 2.2.5 with stringent parameters designed to identify and remove only those read pairs with full-length reference agreement. Reads were mapped to the NCBI GRCh38.7 human genome reference sequence using bowtie2 in global alignment mode (--end-to-end) with low sensitivity (--fast) and stringent output settings (--no-unal --no-mixed --no-discordant). This mapping selected at most one mapping per pair.

Cell line gDNA read pairs that did not map to the human genome reference were assembled per cell line with CLC NGS Cell (Qiagen Bioinformatics) version 3.22.55708 using *de novo* assembly with parameter “-p fb ss 200 400“. Contigs of minimum length 200 were retained and named CLCs to indicate Cell Line Contigs.

The UniVec database was downloaded from the NCBI FTP site (5456 sequences and 1,049,913 bp). Other references were downloaded from GenBank. The reference human assembly was GCA_000001405.22_GRCh38.p7_genomic (3,232,546,710 bp). Mycoplasma sequences were obtained by searching GenBank for Mycoplasma nucleotide sequences of length 500 Kbp or more (263 sequences and 232,800,861 bp). The PhiX genome sequence was NC_001422.1 (5,386 bp). The Sendai RNA reference sequences were AB855653.1 and AB855654.1 (15,384 bp each).

### SDB tests

The RNAseq subtraction step used a sensitive mapping strategy. Although it did not use a splice-aware aligner, it relied on sensitive local alignments, the selection of at most one best alignment per read, and the subtraction of both reads of a pair if either read mapped. The mapping used bowtie2 with parameters “--sensitive-local” and “--no-unal” to map read pairs to the subtraction database SDB1. The mapping step can be parallelized with one pair of input read files per job. Each job requires RAM approximately equal to twice the database size. Our runs against the 3.3 GB SDB1 reserved 8 GB RAM and 4 threads.

RNAseq reads from the Hölzer *et al.* ( Hölzer *et al*., 2016) experiments were kindly provided by the authors (Martin Hölzer, personal communication; NCBI SRA accession SRP128545).

For reads characterized by BLAST, BLASTN 2.2.31+ from the NCBI BLAST+ package was used to search the NCBI nt database. BLAST was run with parameters “-outfmt7 qseqid sseqid pident length mismatch gapopen evalue bitscore staxids” to capture taxon ID and “-max_target_seqs 1” to retain the top hit per query sequence.

## Results

### Construction of the Subtraction Database

To obtain cell line genomic sequence, each cell line was separately subjected to DNA sequencing. For each of three cultured human cell lines, one DNA sequencing library was created with a ~300 bp insert size. Each library was test sequenced on an Illumina MiSeq platform to generate small volumes of 2x300 bp read pairs; in these pairs, reads were expected to overlap. Each library was then sequenced on two runs of an Illumina NextSeq platform to generate high volumes of 2x150 bp read pairs. Reads were trimmed with CutAdapt. The result offered 105 Gbp of sequence in 346 M read pairs, or 28X average coverage of the human genome per cell line. Further details and public accessions are provided in Table S1. The reads were filtered to eliminate pairs that provide sequence that is already present within a standard human genome reference sequence. This was done by mapping the NextSeq genomic read pairs with stringent parameters. This step filtered 94% of read pairs.

To further characterize the mapped reads, the reference mapping was scanned for high-coverage and low-coverage areas. High coverage would indicate sequence present in the cell line at higher copy number than their representation in the reference sequence. For example, coverage over 100X would represent at least 3X higher representation in the cell line than the reference. With this criterion for high coverage, the number of reference bases at high coverage was 8.6 Mbp per cell line. The average high-coverage interval was 106 bp, which is shorter than the 150 bp read length, suggesting that short tandem repeats may be expanded in the cell lines. The mapping was also scanned for low-coverage areas. Low-coverage regions shorter than read pairs would indicate rearrangements within the cell line genomes. The combined span of reference bases with coverage less than 2X was 96 Mbp and the average low-coverage interval was 265 bp. Further details are provided in Table S2.

To capture sequences present in any of the three cell lines, but absent from the reference, the unmapped reads were assembled into Cell Line Contigs (CLCs). The assembly generated about 3 Mbp in 8 K CLCs for each cell line. CLC size statistics are provided in Table S3. The average CLC size was 380 bp though there were a few large CLCs from each cell line. The largest CLC from each cell line had a partial alignment to the largest CLC from the other two lines. Analysis of the two largest contigs per cell line showed similarity to other human or primate sequences in the public databases and redundancy between cell lines ( Table S6).

**Figure 1.**
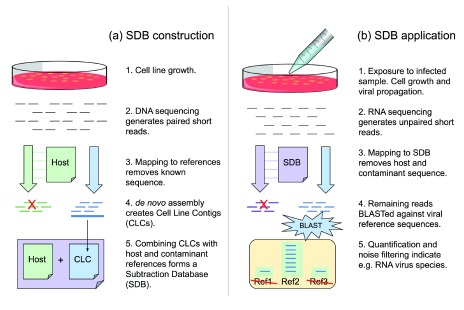
The subtraction database construction and application pipelines. During construction ( **a**), cells are grown without infection. Contigs from cell line gDNA are combined with host and contaminant references to form the subtraction database. During application ( **b**), cells are exposed to an infected sample. RNA virus replication accompanies cell growth. Subsequent RNAseq data is filtered to remove close matches to the subtraction database. Remaining data are characterized by the more costly process of aligning to a reference database.

The subtraction database named SDB1 was constructed by concatenating FASTA representations of the CLCs, the human genome reference, the PhiX genome, the UniVec database, and a collection of Mycoplasma complete genomes. The database construction process is summarized in Figure 1a. Table S4 shows the number of sequences and number of bases per data source.

### Subtraction and detection of Sendai virus

The subtraction database SDB1 was used to filter host sequence from RNAseq data using the process summarized in Figure 1b. The subtraction process was assessed with a test designed to emulate a cell-based RNA virus detection assay. In this assay, environmental samples are analyzed to determine which RNA viruses are present, if any. To overcome the presumed low titer of viral RNA, this assay uses virus-permissive cell lines to amplify viral load. After exposure to environmental samples, the cells in culture are grown for sufficient time for viral replication. RNA is harvested, optionally depleted of rRNA, and sequenced. The rRNA depletion step is employed to enrich the non-host RNA and increase the number of non-host RNAseq reads generated. After sequencing, a sequence analysis step involves alignment of the RNAseq data to reference databases for taxonomic classification and quantification. Classification of host cell reads is uninformative so all computational investment in classifying them represents overhead cost. The goal of the subtraction step is to reduce the overhead without loss of sensitivity. Although the taxon of the virus was known *a priori*, the same process could be used to detect unknown viruses in an uncharacterized sample.

This test used SDB1, our subtraction database that includes genomic DNA sequence derived from the HepG2, HuH-7, and Jurkat human cell lines. This test also used RNAseq from those cell lines. RNAseq reads were generated from multiple samples and mapped to SDB1. This test used uninfected cells and cells infected with the Sendai virus (SeV). After the growth period, cells were treated for rRNA depletion, or left untreated as a control. Twelve libraries were generated in total, representing the three cell lines under four different conditions: infected-and-depleted, infected-and-not-depleted, mock-infected-and-depleted, and mock-infected-and-not-depleted. The libraries were barcoded, multiplexed, and sequenced on a NextSeq platform to generate 2x150 bp read pairs. RNAseq read counts per library are provided in Table S1.

The sequence data were subjected to a subtraction process by mapping to SDB1. The mapping used local alignments to allow host spliced RNA sequences to map to host genomic DNA sequence. The results are shown in Table 1. The read counts after trimming ranged from 19 to 27 million per library ( Table 1, column A). The rate of subtraction ranged from 89% to nearly 100% per library ( Table 1, column B). The relative contribution by each type of SDB1 sequence is shown in Table S5. As expected, the human genome reference sequence subtracted the most reads. The CLCs from the human cell lines subtracted 0.19% to 1.34% of reads per library. Contrary to expectation, the cell line origin of the RNAseq was not predictive of the cell line whose CLCs would subtract the most reads. Instead, the CLCs derived from HuH-7 consistently subtracted more RNAseq reads than those from HepG2 or Jurkat. It is possible that the CLCs derived from HuH-7 capture larger portions of transcriptional units that are partially represented in the other CLCs.

**Table 1.** Enrichment of Sendai virus. Three cell lines were grown with Sendai virus (SeV) infection or mock infection. Some samples were treated with Ribo-Zero (Illumina) to deplete rRNA. (A) All cDNA libraries were sequenced on the Illumina NextSeq platform to generate over 19 million RNAseq reads per sample. (B) At least 89% of reads from every sample mapped to the subtraction database named SDB1. (C) Non-SDB reads were mapped to SeV references. Expressed as a fraction of initial reads, the SeV complement was 5% to 10% in infected samples and less than 1% in the controls. (D) Expressed as a percentage of non-SDB reads, the SeV complement was 78% to 95% in infected-but-not-depleted samples. The complement was larger in the infected-and-depleted samples, and smaller in the mock-infected samples. This suggests that analysis of non-SDB reads could support the detection of known viruses in an uncharacterized sample. The apparent false positive enrichments (e.g. 24.24% SeV in HEP/none/none) can be discounted by applying a minimum requirement for 0.10% viral reads out of initial reads.

			(A)	(B)	(C)	(D)
Cell Line	Virus treatment to cells	rRNA treatment to library	Initial trimmed reads (millions)	Portion of initial mapped to SDB1	Portion of initial mapped to SeV	Portion of non-SDB mapped to SeV
HEP	SeV	depletion	27.20	89.06%	10.38%	96.74%
HEP	SeV	none	22.06	98.96%	0.96%	95.27%
HEP	none	none	24.44	99.94%	0.01%	24.24%
HEP	none	depletion	26.70	99.89%	0.00%	4.01%
						
HUH	SeV	depletion	23.44	91.44%	7.03%	84.14%
HUH	SeV	none	25.79	99.30%	0.51%	77.59%
HUH	none	none	24.72	99.95%	0.00%	2.24%
HUH	none	depletion	20.99	99.91%	0.00%	0.72%
						
JUR	SeV	depletion	26.59	94.18%	5.65%	98.66%
JUR	SeV	none	19.28	99.42%	0.47%	85.77%
JUR	none	none	19.06	99.94%	0.00%	1.68%
JUR	none	depletion	25.10	99.92%	0.00%	1.33%

Next, the RNAseq reads that did not map to SDB1 were extracted for analysis. These “non-SDB” reads were mapped to SeV genome reference sequences. As expected, the libraries with the largest SeV complement were the libraries that had been infected with Sendai virus and depleted of rRNA. When expressed as a portion of initial reads, the SeV complement was 5% to 10% for these RNAseq libraries ( Table 1, column B). When expressed as a portion of the non-SDB reads, the SeV complement was 84% to 99% ( Table 1, column C). This demonstrates that the SDB subtraction step left RNAseq data that was highly enriched for the viral sequence.

In all the datasets derived from infected libraries, SeV was the majority component of the non-SDB reads ( Table 1, column D). This was true even for the non-depleted libraries. When expressed as a portion of initial reads, the SeV complement was 0.47% to 0.96% for non-depleted libraries. When expressed as a portion of non-SDB reads, the SeV complement was 78% to 95% for non-depleted libraries. While the non-depleted libraries did contain fewer SeV reads in absolute terms, the portions of reads identified as SeV after subtraction were almost as high as those for the depleted libraries. This suggests that the SDB subtraction can provide a viable alternative to rRNA depletion for the detection of RNA viruses.

In the datasets from cells that were mock infected, the portion of non-SDB reads mapping to SeV was non-zero ( Table 1, column D). In one case, it was 24%. These results suggest that taxonomic classification by mapping is noisy and that false positive virus identifications could result. Our data suggests a threshold for avoiding false positives, namely that a virus is suspected only if the post-SDB virus read count is as least 0.10% of initial reads. Additional data and additional replicates would assist selection of appropriate thresholds for desired levels of specificity.

The viral taxon was known beforehand and mapping non-SDB reads to SeV references only confirmed *a priori* knowledge. In a discovery situation, the non-SDB reads could be characterized by BLASTn homology search against the NCBI nt database. With our data, SeV was the taxon with the most hits for all libraries. This result indicates that SeV could have been detected *de novo* by BLAST analysis. After SeV, no single taxon was represented at over 0.01% of initial reads. The secondary taxa indicated by BLAST included Newcastle disease virus (concentrated in the one library of HuH-7 with viral infection and depletion), Ebola virus, Human ORFeome Gateway entry vector, *Homo sapiens*, *Gorilla gorilla*, and others. The virus hits are possibly indicators of contamination from simultaneous projects in the lab. The primate hits could be due to the higher sensitivity of the BLAST homology search compared to mapping.

The subtraction computation consumed 1,285 cpu sec per million reads. The BLAST analysis consumed 107,146 cpu sec per million reads. The total cost of subtraction-then-blast was approximately 20% of the hypothetical cost of running BLAST on every read.

### Subtraction and detection of Ebola and Marburg viruses

The utility of SDB1 was tested on data from an independent source. Hölzer *et al.* ( Hölzer *et al*., 2016) explored host cell expression changes in HuH-7 human cells and R06E-J cells from the bat *Rousettus aegyptiacus*. As part of the study, cells were infected with either the Ebola virus strain Zaire, Mayinga (GenBank: NC_002549), or the Lake Victoria Marburg virus, Leiden (GenBank: JN408064.1), or a mock infection. Cells were harvested at 3, 7, or 23 hours after infection and sequenced by RNAseq.

The RNAseq reads from the HuH-7 experiments of Hölzer *et al.* were downloaded and trimmed; see Table 2. From every library, a majority of reads were subtracted by mapping to SDB1 ( Table 2, column B). After subtraction, the non-SDB reads were mapped to reference genome sequences for Ebola (EBOV) and Marburg (MARV). The EBOV complement of the non-SDB reads was 57% to nearly 100% in EBOV-infected libraries and lower in the other libraries ( Table 2, columns C–D). The MARV complement of the non-SDB reads was 39% to 75% in MARV-infected libraries and lower in the other libraries ( Table 2, columns E–F). These results confirm that SDB1 can provide effective enrichment of other viruses within independent cell cultures of HuH-7.

**Table 2.** Enrichment of Ebola and Marburg viruses. Data from an independent study ( Hölzer *et al*., 2016) are derived from RNAseq of Huh-7 cells infected with Ebola virus (EBOV), Marburg virus (MARV), or none (Mock). The data were re-analyzed here using SDB1. Subtraction enriched the EBOV complement to at least 57% in the EBOV-infected samples. Subtraction enriched the MARV complement to at least 39% in the MARV-infected samples. The apparent false positive enrichments can be discounted by applying a minimum requirement for 0.10% viral reads out of initial reads.

		(A)	(B)	(C)	(D)	(E)	(F)
Virus	Time	Initial trimmed reads (millions)	Portion of initial mapped to SDB1	Portion of initial mapped to EBOV	Portion of non-SDB mapped to EBOV	Portion of initial mapped to MARV	Portion of non-SDB mapped to MARV
EBOV	03h	32.87	99.70%	0.16%	57.17%	0.00%	0.12%
EBOV	07h	46.62	97.47%	2.34%	94.41%	0.00%	0.01%
EBOV	23h	50.41	62.18%	37.29%	99.56%	0.00%	0.00%
							
MARV	03h	41.81	99.76%	0.01%	3.62%	0.08%	38.96%
MARV	07h	45.81	99.06%	0.03%	3.03%	0.63%	69.49%
MARV	23h	34.37	97.61%	0.02%	0.87%	1.71%	75.45%
							
Mock	03h	38.55	99.82%	0.02%	10.79%	0.00%	0.20%
Mock	07h	36.03	99.86%	0.01%	7.59%	0.00%	0.18%
Mock	23h	36.99	99.80%	0.02%	9.84%	0.00%	0.16%

## Discussion

The establishment of host sequence databases from commonly used cell lines, especially for lines often used to propagate viruses, is a critical control to ensure experimental results are attributed to the specific virus being tested. Therefore, we analyzed the human cell lines HepG2, HuH-7, and Jurkat in order to increase their utility for amplifying and detecting viruses in clinical or environmental samples. We developed a subtraction database ( Daly *et al*., 2015) for the computational removal of host reads from RNAseq datasets. The database consists of the GRCh38 reference human genome sequence plus cell line specific sequences and potential contaminant sequences. The database contained DNA sequence from uninfected cells. We sequenced genomic DNA, rather than mRNA, in order to capture cell line genes whose expression may be limited to the viral propagation stage. We demonstrated utility by mapping RNAseq reads from cells infected with RNA viruses, as well as control cells, to the database. We were able to subtract host sequences and enrich each dataset for the non-host complement.

The subtraction process reduced the computational cost that would be incurred by characterization of every read. The subtraction process removed host reads which often represented 99% of the data. The subtraction process used mapping software which imposed approximately 1% of the computational cost compared to characterization by BLAST. However, the identification of known sequences could be accomplished at lower cost using alignment-free K-mer matching software such as Kraken ( Wood & Salzberg, 2014). Our subtraction process relied on the Bowtie2 ( Langmead & Salzberg, 2012) mapping software. The process could be modified to use instead a splice-aware aligner such as HiSat2 ( Kim *et al*., 2015). Whereas our highly sensitive process subtracts all read pairs with even one partial alignment, a splice-aware process might achieve higher specificity by subtracting only those RNA pairs with full-length alignments including spliced alignments. Being a faster mapper, HiSat2 might also reduce the computational cost of subtraction.

Our subtraction database included novel sequences specific to the three cell lines. Since it is not yet economical to generate complete genome and transcriptome assemblies of each subject cell line, we generated short reads at low coverage and retained only those reads that did not map to the human reference. Subsequent *de novo* assembly of the retained reads yielded 8 Mbp of novel sequence per cell line. The resulting cell line contigs (CLCs) were included in the subtraction database.

The marginal value of the CLCs was low. The CLCs were responsible for only 0.19% to 1.34% of RNAseq subtraction per sequencing experiment. It thus appears that the cost of CLC construction outweighed the benefit, at least for the cells and viruses tested. Investigators constructing subtraction databases for other human cell lines should evaluate the utility of existing public sequences prior to cell line sequencing. CLCs could be more beneficial on cell lines harboring transcribed endogenous viral sequence, on cell lines derived from non-human species for which prior sequences are lacking, on human cancer cell lines harboring divergent genomes, and on cells suspected of harboring transfection-induced chromosomal rearrangements, as was reported for transfected HepG2 cells ( Livezey *et al*., 2002). CLCs could inform the search for marker sequences for cell line authentication ( Almeida *et al*., 2016).

Characterization of the CLCs remains for future work. The largest CLCs had full-length alignments to complete sequences of clones from chromosomes of human or other primates, ( Table S6) and partial alignments to CLCs from the other cell lines used here. Our coverage analysis of reads that did map to the reference suggested copy number variation and structural variation in the cell line genomes. It is likely that many CLCs capture cell-line specific chromosomal breakpoints such as those reported in HepG2 ( Wong *et al*., 2000) or cell-line specific retrotransposed insertions. 

## Data availability

The sequencing reads are available in NCBI with these SRA accessions: HepG2 DNA (SRR5296488, SRR5296494, SRR5296491) and RNA (SRR5296490, SRR5296492, SRR5296493, SRR5296495), HuH-7 DNA (SRR5297887, SRR5297975, SRR5297924) and RNA (SRR5297992, SRR5297976, SRR5297993, SRR5297994), and Jurkat DNA (SRR5294049, SRR5293982, SRR5293981) and RNA (SRR5293979, SRR5293983, SRR5295385, SRR5293984). Hyperlinked BioSample accessions are listed in Table S1. The subtraction database is available at GitHub ( JCVenterInstitute): https://github.com/JCVenterInstitute/HumanSubtractionDB1/blob/master/SDB1.fasta.gz

Archived scripts as at time of publication: http://doi.org/10.5281/zenodo.1146104 ( Miller *et al*., 2018)

License: GNU GPL v3.0

## Supplementary material

The following are provided as an Excel workbook:

Table S1: Characteristics and accessions of human cell line sequencing data.

Table S2: Human reference coverage by cell line reads.

Table S3: Size statistics for Cell Line Contigs (CLCs).

Table S4: Composition of the SDB.

Table S5: Contribution per cell line.

Table S6: Blast characterization of the largest CLCs per cell line.

Table S7: Top blastn hits for JCVI nonSDB reads.
