# Supplementary material for: A host subtraction database for virus discovery in human cell line sequencing data
Source: F1000Res. 2019 May 21;7:98. Originally published 2018 Jan 23. [Version 3] doi: 10.12688/f1000research.13580.3 (PMC6556987; doi:10.12688/f1000research.13580.3)
Supplement: Peer review history

# Peer review: Reviewer response for version 3

**DOI:** 10.5256/f1000research.21227.r48880

**Published:** 2019-06-04

**Authors:** Ravi Sachidanandam

**Affiliations:** 1 Department of Oncological Sciences, The Tisch Cancer Institute, Icahn School of Medicine at Mount Sinai, New York, NY, USA

The figure helps immensely in understanding what was done. The clarifications have also made it easier to understand the scope of the work and its utility.

I have read this submission. I believe that I have an appropriate level of expertise to confirm that it is of an acceptable scientific standard.

# Peer review: Reviewer response for version 2

**DOI:** 10.5256/f1000research.16922.r45082

**Published:** 2019-03-20

**Authors:** Ravi Sachidanandam

**Affiliations:** 1 Department of Oncological Sciences, The Tisch Cancer Institute, Icahn School of Medicine at Mount Sinai, New York, NY, USA

I offer some brief comments that could be addressed to make the paper more accessible and also justify the work.

1. A schematic of the process used in the study. There was a very detailed description that could have been shortened by providing a schematic. This would enhance the paper, by making it easier to read and understand.
2. What is the marginal advantage of the data in the paper? If one were to just use mapping to the reference human genome (hg38), I assume most of the host-derived reads would get eliminated. How many more reads are eliminated using the database provided in this paper? If this is not a big number, then maybe it is not worth the work?
3. There were some contigs assembled that do not map to the reference human genome? What are these? Some hints on where they come from?
4. Is the DNA or mRNA data most useful? It was not clear to me at the end if one or the other was more useful in the subtraction.

I have read this submission. I believe that I have an appropriate level of expertise to confirm that it is of an acceptable scientific standard, however I have significant reservations, as outlined above.

# Peer review: Reviewer response for version 2

**DOI:** 10.5256/f1000research.16922.r36005

**Published:** 2018-07-30

**Authors:** Caroline C. Friedel

**Affiliations:** 1 Department of Computer Science, Ludwig Maximilian University of Munich, Munich, Germany

The authors appropriately addressed my concerns and I have no further comments to make.

I have read this submission. I believe that I have an appropriate level of expertise to confirm that it is of an acceptable scientific standard.

# Peer review: Reviewer response for version 1

**DOI:** 10.5256/f1000research.14751.r30576

**Published:** 2018-03-19

**Authors:** Caroline C. Friedel

**Affiliations:** 1 Department of Computer Science, Ludwig Maximilian University of Munich, Munich, Germany

In this article, the authors present a host subtraction database for human cell line sequencing. The purpose of this approach is subtract non-viral reads vom RNA-seq samples of virus-infected cells to improve efficiency of subsequent analyses. Their substraction database is targeted specifically towards three commonly used human cell lines: HepG2, HuH-7 and Jurkat.

 In brief, their approach was as follows:

 For creation of the host subtraction database (SDB):

 - Perform DNA sequencing for the cell lines considered

 - Align reads to the human reference genome

 - Assemble unaligned reads into contigs

 The SBD was then created as a concatenation of FASTA sequences of the assembled contigs, the human reference genome, the UniVec Database (nucleic acid sequences which may be of vector origin), the PhiX genome (used as control in Illumina sequencing) and a collection of Mycoplasma genomes

 The subtraction process was then performed by aligning reads with bowtie2 against the SDB and then removing all read pairs if at least one read in the pair could be mapped with a local alignment to the SDB.

 Evaluation of their approach was performed using RNA-seq data of the three cell lines either infected or not infected with Sendai Virus (SeV) as well as Huh-7 cells infected with Ebola or Marburg virus or not infected. They show that after host subtraction, virus reads make up the largest portion of the remaining reads. They also show that the combined runtime of first running bowtie2 and then BLAST on the remaining reads, e.g. to identify the virus, is only around 20% of the time that would be required if BLAST were run on all reads.

 While the article is well written and the method is well described, there are a number of issues that need to be addressed:

 1) The advantage of adding contigs assembled from DNA sequencing to the SDB is relatively small. Only between 0.19% and 1.34% of reads were subtracted in the SeV test case. However, the effort for obtaining these contigs is substantial as it requires DNA sequencing and contig assembly. Thus, the SDB cannot be easily extended to other cell lines without additional experiments. To show that this additional effort is warranted, the authors should at least show the following:

- That removing reads mapping to the contigs before post SDB mapping has a substantial influence on the results by either resulting in reduced numbers of false positive mappings to the Sendai genome or substantially reducing runtime of the total pipeline (including SDB mapping and potentially BLAST analysis).
- That a similar effect cannot be obtained by augmenting the human genome by publicly available human sequence data not part of the reference genome, e.g. transcript sequences from Ensembl or RefSeq or alternatively publicly available RNA-seq data for the considered cell lines. In particular, the latter might even outperform DNA sequencing for the cell lines as assembly of reads not mapping to the human genome would result in novel transcript sequences to which RNA-seq reads map better with the (unspliced) read aligner used in this approach. Furthermore, it would allow easily extending the SDB approach to other cell lines for which RNA-seq data already is/becomes available.

2) There should be some justification for the use of an unspliced read aligner, i.e. bowtie2, for mapping RNA-seq reads against the SDB, rather than a dedicated RNA-seq mapping program that also allows identifying spliced read alignments, such as STAR or HISAT2. In particular, the latter is both fast and requires relatively little memory. While removing reads for which bowtie2 identifies a local alignment likely identifies all sequences originating from the SDB sequences, there is a potential that too many reads may be removed, e.g. in case of local similarities between SDB sequences and the virus in consideration. A safer approach would be to only exclude reads that can be aligned completely or almost completely (including gaps due to splicing). Regarding this point, the authors should show that:

- there is a substantial speedup of using local alignment with bowtie2 compared to fast RNA-seq alignment with HISAT2 or STAR
- that results do not differ much between the two approaches, i.e. the bowtie2 approach does neither miss many mappings a dedicated RNA-seq mapper would find nor reports many mappings not found using an RNA-seq mapper. If the latter is the case, they should show that these additional mappings missed by an RNA-seq mapper are valid mappings to the human cell lines and influence subsequent results.

Finally, DNA and RNA sequencing data for the considered cell lines should be submitted to a public database to ensure full reproducibility of their approach.

I have read this submission. I believe that I have an appropriate level of expertise to confirm that it is of an acceptable scientific standard, however I have significant reservations, as outlined above.

# Response

**Published:** 2018-07-03

**Authors:** Jason Miller

**Affiliations:** Shepherd University, USA

*Reviewer Comment #1.*

*This comment is focused on the cell line contigs (CLCs) that were constructed and added to the subtraction database (SDB). It notes that the CLC construction investment yielded relatively little benefit. It says the manuscript should contain at least two follow-up experiments to measure the effectiveness of SDBs constructed with and without CLCs. The additional experiments might inform those constructing SDBs on other cell lines.*

We agree that the manuscript should clearly convey the finding that the CLCs offered little advantage on these cells and these viruses. The manuscript already noted that the CLCs subtracted only 0.19% to 1.34% of RNA reads, providing “a small amount of mapping sensitivity.” However, the extensive treatment of CLCs in the Discussion seemed to emphasize their role. We deleted almost three paragraphs about CLCs from the Discussion. We inserted what is now the third paragraph of Discussion which includes the statement, “ **It thus appears that, for the cells and viruses tested, the cost of CLC construction outweighed the benefit.**”

With this change incorporated, it is important to note the Huh7, HepG2, and Jurkat cell lines are commonly used for viral propagation. Cell lines often have genomic abnormalities not previously reported in public databases. From this standpoint, generation of sequence data and the corresponding CLCs provides a valuable reference dataset to the community, if only to show consistency with public domain data. The first sentence of the revised Discussion now says host sequence databases provide “ **a critical control to ensure experimental results are attributed to the specific virus being tested.**”

One can question whether an essentially equivalent resource could be reproduced at lower cost. Performing that experiment would go beyond the scope of this F1000Resarch paper, which announces an existing resource that readers may download without charge. The question is certainly relevant for construction of future subtraction databases. Therefore, we modified the manuscript to raise the issue. The new third paragraph of Discussion includes the statement, “ **Investigators constructing subtraction databases for other human cell lines should evaluate the utility of existing public sequences prior to cell line sequencing.**” It also lists types of cell lines for which sequencing could reasonably provide more benefit than it did for us.

*Reviewer Comment #2.*

*This comment is focused on the mapping of RNA reads to genomic sequences. It says the process may remove too many reads because the Bowtie2 mapper is not splice-aware. It says the manuscript should demonstrate that Bowtie2 performs better than alternatives such as HiSat2.*

Our process employs high sensitivity by design. It uses Bowtie2 parameterized for sensitive alignments and it subtracts both RNA reads of any pair that shows any detectable alignment, including pairs with only one partial alignment to one read. We considered HiSat2 during the design phase, but we reasoned that a HiSat2-based process would be less sensitive if it only subtracted reads with partial alignments explained by splicing. One could probably design and build several alternate processes, test them on our data, and characterize all the reads that subtract differentially. Such testing might be informative, but it would go beyond the scope of this F1000Research manuscript, which describes one process that is already functional, characterized, and available. Since many readers may wonder about our choice of mapper, we modified the manuscript to point out that one could implement a splice-aware variant of our process. The new text, added to the second paragraph in the revised Discussion, says, “ **Our subtraction process relied on the Bowtie2 mapping software. The process could be modified to use instead a splice-aware aligner such as HiSat2. Whereas our highly sensitive process subtracts all read pairs with even one partial alignment, a splice-aware process might achieve higher specificity by subtracting only those RNA pairs with full-length alignments including spliced alignments. Being a faster mapper, HiSat2 might also reduce the computational cost of subtraction.**”

*Reviewer Comment #3.*

*This comment is focused on data availability of sequence reads.*

All of the DNA and RNA reads were previously deposited at NCBI but the fact was easy to miss with accessions being given in the supplement only. The revised text says, “ **The sequencing reads are available at NCBI with these SRA accessions: HepG2 DNA (SRR5296488, SRR5296494, SRR5296491) and RNA (SRR5296490, SRR5296492, SRR5296493, SRR5296495), HuH-7 DNA (SRR5297887, SRR5297975, SRR5297924) and RNA (SRR5297992, SRR5297976, SRR5297993, SRR5297994), and Jurkat DNA (SRR5294049, SRR5293982, SRR5293981) and RNA (SRR5293979, SRR5293983, SRR5295385, SRR5293984).”**
